# Transgenerational Inheritance of Increased Fat Depot Size, Stem Cell Reprogramming, and Hepatic Steatosis Elicited by Prenatal Exposure to the Obesogen Tributyltin in Mice

**DOI:** 10.1289/ehp.1205701

**Published:** 2013-01-15

**Authors:** Raquel Chamorro-García, Margaret Sahu, Rachelle J. Abbey, Jhyme Laude, Nhieu Pham, Bruce Blumberg

**Affiliations:** 1 Department of Developmental and Cell Biology, and; 2 Department of Pharmaceutical Sciences, University of California, Irvine, Irvine, California, USA

**Keywords:** adipogenesis, endocrine disruption, MSCs, NAFLD, nonalcoholic fatty liver disease, obesogen, PPARγ, TBT, transgenerational, tributyltin

## Abstract

Background: We have previously shown that exposure to tributyltin (TBT) modulates critical steps of adipogenesis through RXR/PPARγ and that prenatal TBT exposure predisposes multipotent mesenchymal stem cells (MSCs) to become adipocytes by epigenetic imprinting into the memory of the MSC compartment.

Objective: We tested whether the effects of prenatal TBT exposure were heritable in F2 and F3 generations.

Methods: We exposed C57BL/6J female mice (F0) to DMSO vehicle, the pharmaceutical obesogen rosiglitazone (ROSI), or TBT (5.42, 54.2, or 542 nM) throughout pregnancy via the drinking water. F1 offspring were bred to yield F2, and F2 mice were bred to produce F3. F1 animals were exposed *in utero* and F2 mice were potentially exposed as germ cells in the F1, but F3 animals were never exposed to the chemicals. We analyzed the effects of these exposures on fat depot weights, adipocyte number, adipocyte size, MSC programming, hepatic lipid accumulation, and hepatic gene expression in all three generations.

Discussion: Prenatal TBT exposure increased most white adipose tissue (WAT) depot weights, adipocyte size, and adipocyte number, and reprogrammed MSCs toward the adipocyte lineage at the expense of bone in all three generations. Prenatal TBT exposure led to hepatic lipid accumulation and up-regulated hepatic expression of genes involved in lipid storage/transport, lipogenesis, and lipolysis in all three subsequent generations.

Conclusions: Prenatal TBT exposure produced transgenerational effects on fat depots and induced a phenotype resembling nonalcoholic fatty liver disease through at least the F3 generation. These results show that early-life obesogen exposure can have lasting effects.

Emerging evidence supports the idea that prenatal and early postnatal events such as maternal nutrition and drug and chemical exposure are manifested in health consequences later in life (reviewed by Barker 2007; Janesick and Blumberg 2011). Obesity has become a burgeoning epidemic in the developed world over the past 30 years (Flegal et al. 2010). Obesity, together with other metabolic risk factors such as insulin resistance, hypertension, and nonalcoholic fatty liver disease (NAFLD), is strongly correlated with the development of cardiovascular disease and diabetes. The total cost of obesity-related disorders has been estimated at $208 billion (in 2008 dollars), or approximately 21% of health care costs in the United States alone (Cawley and Meyerhoefer 2012). It is widely believed that an increase in fat mass occurs as the primary result of impaired balance between energy intake and energy expenditure; however, additional factors are important contributors to obesity. These include smoking, stress, sedentary lifestyle, excessive consumption of alcohol, and genetics (Garruti et al. 2008; Herbert 2008; Hill and Peters 1998; Power and Jefferis 2002; Rippe and Hess 1998). Epidemiological studies in human populations have demonstrated a correlation between parental obesity and the development of obesity in their children during childhood (Danielzik et al. 2002; Power et al. 1997). Although little is known about the underlying mechanisms, one recent study demonstrated a correlation between the epigenetic state of DNA from the umbilical cord of children and adiposity later in their childhood (Godfrey et al. 2011). There are currently no studies that have examined whether such epigenetic modifications can be transmitted to subsequent generations in humans.

Recent work from our laboratory and from other laboratories supports a role for environmental factors in the development of obesity; these factors include exposure, especially during critical developmental windows, to endocrine-disrupting chemicals (EDCs) (reviewed by Heindel 2011; Janesick and Blumberg 2011; La Merrill and Birnbaum 2011; Newbold et al. 2009; Tang-Peronard et al. 2011). “Obesogens” are chemicals that promote obesity directly by increasing adipocyte size and/or number, or indirectly by altering metabolic set points or interfering with the regulation of appetite and satiety. Our environmental obesogen hypothesis proposes that a subset of EDCs could promote the development of obesity. Although initially controversial, the obesogen hypothesis has gained momentum in recent years with the identification of obesogenic chemicals that promote adipogenesis and obesity in animals and humans (reviewed by Janesick and Blumberg 2011). In previous studies, we identified tributyltin (TBT) as an environmental obesogen. Prenatal TBT exposure increased adipose depot size in mice via activation of peroxisome proliferator activated receptor γ (PPARγ) (Grün et al. 2006; Li et al. 2011) and reprogrammed the fate of multipotent mesenchymal stromal stem cells (MSCs) to favor the adipogenic lineage at the expense of the osteogenic lineage, both *in vitro* and *in vivo* (Kirchner et al. 2010).

A growing body of evidence shows that developmental exposure to EDCs leads to adverse health outcomes later in life (reviewed by Diamanti-Kandarakis et al. 2009). EDCs can act directly on nuclear hormone receptors, on a variety of transcriptional cofactors, on enzymatic pathways involved in hormone biosynthesis or metabolism, and on neuroendocrine signaling pathways. Many effects of EDC exposure are manifested as disturbances of endocrine or reproductive systems (Diamanti-Kandarakis et al. 2009), and recent evidence suggests that some of these phenotypes can be transmitted to subsequent generations (Anway et al. 2005, 2008; Blatt et al. 2003; Goncalves et al. 2010; Nilsson et al. 2012; Wolstenholme et al. 2012; Zambrano et al. 2005). Until now, it was unknown whether the obesity-related effects of obesogen exposure were heritable in a multi- or transgenerational manner.

In the present study, we exposed pregnant F0 mice to three different concentrations of TBT (5.42, 54.2, or 542 nM) in the drinking water—concentrations that deliver approximately 50-fold lower, 5-fold lower, and 2-fold higher doses, respectively, compared with the established no observable adverse effect level (NOAEL) of 25 μg/kg/day (Vos et al. 1990). F1 animals were exposed directly during *in utero* development, and F2 animals were potentially exposed as germ cells of the F1. Effects noted in F1 and F2 generations are termed “multigenerational” (Skinner 2008). F3 animals are the first generation that received no exposure to TBT at any time, and phenotypes observed in F3 animals are considered to be transgenerational and permanent (Anway and Skinner 2006; Jirtle and Skinner 2007).

We sought to determine whether exposure of pregnant F0 mice to TBT would lead to effects on exposed F1 animals as well as on their F2 and F3 descendents. Compared with controls, offspring of mice exposed prenatally to TBT (F2) and their descendants (F3) exhibited increased adipose depot weight, larger adipocyte size, increased adipocyte number, and biased cell fate in the MSC compartment to favor the adipocyte lineage at the expense of the bone lineage. These changes occurred despite animals eating a normal diet. TBT-exposed animals and their descendants developed fatty livers and exhibited altered hepatic gene expression suggestive of NAFLD in all three generations. Our results demonstrate that the effects of prenatal TBT exposure are permanent and transgenerational, suggesting an increased risk to future generations of developing obesity and related disorders, such as NAFLD. These results have important implications for the ongoing debate about developing appropriate policies to minimize the negative effects of EDC exposure.

## Materials and Methods

*Animals.* Male and female C57BL/6J mice (8 weeks of age) were purchased from The Jackson Laboratory (Bar Harbor, ME). Mice were housed in micro-isolator cages in a temperature-controlled room (22–24°C) with a 12-hr light/dark cycle and provided water and food (standard low-fat diet for rodents RMH 2500; Purina Mills, Richmond, IN) *ad libitum*. Animals were treated humanely and with regard for alleviation of suffering. All procedures conducted in this study were approved by the Institutional Animal Care and Use Committee of the University of California, Irvine.

*Fetal exposure to TBT*. Female C57BL/6J mice (six females per treatment group) were exposed, via drinking water, to TBT (5.42, 54.2, or 542 nM), 500 nM rosiglitazone (ROSI; a pharmaceutical obesogen), or DMSO vehicle (all of which were diluted in 0.5% carboxymethyl cellulose in drinking water to maximize solubility) during the 7 days before mating. Plug detection was defined as embryonic day 0.5. Chemical treatment was provided to the females throughout pregnancy, so F1 animals were exposed to the chemicals during *in utero* development and F2 animals were exposed as germ cells in the F1 mice. F3 animals were not exposed to the chemicals. F1 and F2 animals from different litters within each exposure group were mated to each other, avoiding sibling inbreeding (six females and six males per exposure group). We analyzed only litters that had five to seven pups, so the analysis included four or five litters per exposure group (see Supplemental Material, Table S1 (http://dx.doi.org/10.1289/ehp.1205701)]. No statistically significant differences were observed in the number of pups per litter among the different groups, and we considered both male and female offspring in our analysis. Mice were euthanized by cervical dislocation at 8 weeks of age (8–16 animals/group; see Supplemental Material, Table S2).

*Histology and DNA isolation.* We isolated and weighed epididymal/ovarian, perirenal, and interscapular white (WAT) and brown (BAT) adipose tissues. These tissues were divided into two groups: one to be prepared for histological analysis and the other for DNA isolation.

Adipose tissue samples for histological analysis were fixed in 3.7% formaldehyde, embedded in paraffin, sectioned, and stained with hematoxylin and eosin (H&E), following standard protocols, in the core facility of the Department of Pathology and Laboratory Medicine at the University of California, Irvine. We used a Zeiss Axiovert 40 CFL microscope (40× magnification) to acquire bright field photomicrographs. Adipocyte size was measured using Adobe Photoshop CS4 (Adobe Systems Inc., San Jose, CA). Adipocyte areas were selected and measured, and the area expressed as the number of pixels per selected area; measurements for 100 cells from each animal were averaged. BAT lipid content is shown as the percentage of the area covered by lipid vesicles, measured using ImageJ (Schneider et al. 2012).

For DNA isolation, 1 mL RLT buffer (Qiagen) was added to 20 μg of each adipose tissue sample. Tissues were homogenized with a motor-driven micropestle. DNA quantitation was performed using Quanti-it™ PicoGreen® dsDNA Reagent (Invitrogen, Carlsbad, CA) as previously described by Kanno et al. (2006).

The left lobe of the liver of each animal was isolated, fixed in 3.7% formaldehyde, kept at 4°C overnight. They were washed with 1 × PBS for 24 hr, and held in 30% sucrose. Tissues were then embedded in OCT compound, flash frozen, and stored at –20°C for subsequent sectioning (10 μm) in a cryostat. After liver sections were rinsed with distilled water and then in 60% isopropanol in distilled water, they were stained for 20 min with Oil Red O (4 g/L in 60% isopropanol). The slides were washed with 60% isopropanol to avoid Oil Red O precipitation and then washed with 1 × PBS for 5 min; samples were counterstained with hematoxylin. We analyzed at least five liver sections per exposure group, and a representative photomicrograph was selected for the final figure. Differential interference contrast (DIC) photomicrographs were acquired on a Zeiss Axioplan II microscope (40× magnification).

*Mesenchymal stem cell isolation*. Bone marrow mesenchymal stem cells (MSCs) were isolated from femurs and tibia by flushing them with media and expanding them in Dulbecco’s modified Eagle’s medium (DMEM) supplemented with 10% calf bovine serum, 2 mM sodium pyruvate, 100 IU/mL penicillin, and 100 μg/mL streptomycin. Cells were maintained in subconfluent culture as previously described (Chamorro-García et al. 2012).

*Quantitative real-time reverse transcriptase polymerase chain reaction (QPCR).* Total RNA was extracted from MSCs and livers using TRIzol reagent (GIBCO-BRL, Gaithersburg, MD). Complementary DNA was generated from 2 μg DNase-treated RNA using Transcriptor Reverse Transcriptase (Roche, Nutley, NJ) following the manufacturer-recommended protocol. Real-time RT-PCR was performed in the DNA Engine Opticon Thermal Cycler (MJ Research/Bio-Rad Laboratories, Hercules, CA). QPCR was performed using FastStart SYBR Green Master Mix (Roche) and 100 nM of primers [see Supplemental Material, Table S3 (http://dx.doi.org/10.1289/ehp.1205701)]. Primer3 software (Rozen and Skaletsky 2000) was used to select primers, and we verified that each primer produces a single peak by gel electrophoresis and melting curve analysis. Target genes examined were *PPAR*γ2, *PPAR*α, fatty acid binding protein 4 (*Fabp4*), zinc finger protein 423 (*Zfp423*), lipoprotein lipase (*LPL*), preadipocyte factor-1 (*Pref-1*), alkaline phosphatase (*ALP*), runt-related protein 2 (*Runx2*), steroid receptor element binding protein 1c (*SREBP1c*), glycerol kinase (GyK), fatty acid synthase (*FASN*), acyl-CoA oxidase (*ACOX*), fat specific protein 27 (*Fsp27/CideC*), fatty acid transporter protein (*FATP*). We quantified expression levels of each target gene relative to β-actin (housekeeping gene) in the same sample following the 2^–ΔΔC_T_^ method (Livak and Schmittgen 2001).

*Statistical analysis*. Data are presented as mean ± SEM. One-way analysis of variance (ANOVA) with Dunnett’s post hoc test was used to determine significant differences in measured outcomes among fat depot weight, adipocyte size, adipocyte number, and body weights from different exposure groups. *p* < 0.05 was considered statistically significant. We found no statistically significant variation in litter representation among DMSO, ROSI, or TBT exposure groups or across generations (DMSO vs. TBT, *p* = 0.2743, 0.3965, and 0.5174 for F1, F2, and F3, respectively); therefore, we analyzed tissues from individual animals rather than pooling data from litters. Unpaired *t*-tests were used for QPCR analysis. We used GraphPad Prism 5.0 (GraphPad Software Inc., San Diego, CA) to perform the statistical analysis.

## Results

*TBT exposure elicits transgenerational effects on adipose depot weight, adipocyte size, and adipocyte number*. A previous study demonstrated epigenetic changes in *PPAR*γ target gene expression and stable changes in MSC programming (Kirchner et al. 2010). Therefore, we examined whether the effects of prenatal TBT exposure could be passed on to subsequent generations. Female C57BL/6J mice were treated prior to conception and throughout pregnancy with vehicle (DMSO), the pharmaceutical obesogen ROSI, or the environmental obesogen TBT. The F1 offspring of these mice (exposed *in utero*), from different litters, were bred to produce F2 mice (potentially exposed as germ cells). F2 mice were then bred to yield F3 animals. F1 animals were directly exposed *in utero* to TBT, whereas F2 animals were potentially exposed as germ cells within the embryonic F1 animals. The F3 generation was never exposed to the chemicals, and any changes observed in F3 are considered to be transgenerational and permanent (Anway and Skinner 2006; Jirtle and Skinner 2007).

We analyzed overall body weight, fat depot weight, MSC gene expression, hepatic lipid accumulation, and hepatic gene expression in male and female offspring at 8 weeks of age (the maximum age from which we could reliably prepare viable MSCs). To ascertain whether changes in fat depot weight were due to hypertrophy, hyperplasia, or both, we measured adipocyte size and the depot DNA content per milligram of tissue to determine adipocyte number in all isolated WAT and BAT. Two visceral depots were sampled: the epididymal/ovarian WAT and the perirenal WAT. The subcutaneous depot analyzed was the interscapular WAT. We also dissected BAT away from the interscapular WAT for separate analysis.

**Figure 1:**
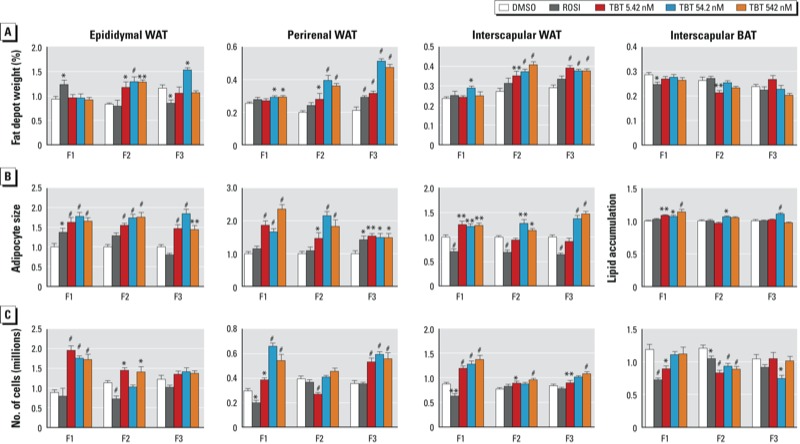
Transgenerational effect of DMSO (vehicle), ROSI, or TBT (5.42, 54.2, or 542 nM) on adipose depots (epididymal WAT, perirenal WAT, interscapular WAT, and interscapular BAT) from F1, F2, and F3 male mice. (*A*) Adipose tissue weights are represented as the percentage of total body weight. (*B*) Relative adipocyte size in epididymal, perirenal, and subscapular WAT (expressed as the number of pixels per selected area, with measurements for 100 cells/animal), and lipid accumulation in subscapular BAT (expressed as a percentage of the area covered by lipid vesicles). (*C*) Number of adipocytes per fat depot as assayed by total DNA quantitation. All data are expressed as the mean ± SEM of 9–18 animals per exposure group. *p < 0.05, **p < 0.01, and ^#^*p* < 0.001 compared with DMSO vehicle by one-way ANOVA with Dunnett’s post hoc test.

We observed striking increases in WAT depots of TBT-exposed animals. In TBT-exposed F1 males, we observed significant increases in the weights of two of three WAT depots (perirenal and interscapular) and substantial increases in adipocyte size and number, particularly in visceral WAT depots. [Figure 1; see Supplemental Material, Figure S1 (http://dx.doi.org/10.1289/ehp.1205701) for representative sections]. The increased number and size of adipocytes in F1 males was not completely reflected in the overall depot weights, which showed modest increases, suggesting that there may be a difference in the density of lipids stored in the WAT of TBT-exposed animals (Figure 1). The effects on fat depot weights in F2 males were more marked than in F1 males, with substantial and significant increases in all visceral WAT depots from offspring of TBT-exposed animals (all TBT doses), whereas the subcutaneous (interscapular) WAT depot showed effects only at the two highest doses of TBT (Figure 1A). In contrast to TBT, we observed little change in WAT depot weights in ROSI-exposed mice. In ROSI-exposed F1 males, the epididymal WAT depot weight increased, whereas adipocyte size was increased but the number of cells did not change; other WAT depots showed no increases in weight and no change to decreased adipocyte size and number. Adipocyte size (Figure 1B) and number (Figure 1C) were increased in all F2 WAT depots from the TBT exposure groups. F3 males from the TBT groups also showed significant increases in the weights of all WAT depots as well as in adipocyte size and number (although not in all depots at every dose) (Figure 1), demonstrating that the effects of prenatal TBT exposure were fully transgenerational in males.

Females showed more modest changes in fat depot size, although most doses of TBT led to significant increases in WAT depot weight and adipocyte size in F1 and F2 animals [see Supplemental Material, Figure S2A,B (http://dx.doi.org/10.1289/ehp.1205701)]. The number of cells in the ovarian WAT depot was decreased in F1 and F3 animals but was increased in the perirenal and interscapular depots of F1, F2, and F3 animals (although not at all TBT doses) (see Supplemental Material, Figure S2C). The perirenal depot increased in size in F3 females, but there were no increases in ovarian and interscapular WAT in F3 animals at 8 weeks of age. However, adipocyte size was elevated in visceral WAT but was slightly decreased or unchanged in interscapular WAT (see Supplemental Material, Figure S2B). Adipocyte number was decreased (ovarian) to slightly increased (perirenal, interscapular) in the WAT of F3 females (see Supplemental Material Figure S2C).

In contrast to the strong transgenerational effects on WAT depot size, effects on body weight in both males and females were less pronounced in these 8-week-old animals. F1 animals showed very modest effects in body weight, with ROSI exposure leading to a slight increase in males and exposure to the highest dose of TBT (542 nM) leading to a slight (but not statistically significant) decrease in females [see Supplemental Material, Figure S3 (http://dx.doi.org/10.1289/ehp.1205701)]. Animals in the TBT exposure groups exhibited significant differences in body weight in F2 males but not females. F3 males showed a slight decrease in body weight at the lowest dose of TBT (5.42 nM), and females showed a slight increase at 54.2 and 542 nM. Overall, we conclude that the effects on body weight at 8 weeks of age are modest, except for F2 males, probably because these animals are young and were maintained on normal chow.

Effects on depot size, adipocyte size, and the number of cells in BAT were modest in both males and females. We observed a trend toward decreased BAT weight in all three generations of both males and females that rarely reached statistical significance in TBT-exposed animals [Figure 1A; see also Supplemental Material, Figure S2A (http://dx.doi.org/10.1289/ehp.1205701)]. There was an accompanying increase in the area covered by lipid vesicles in F1, F2, and F3 males (although not in all TBT exposure groups) and in F1 females (Figure 1B; see also Supplemental Material, Figure S2B). Histological analysis showed large vesicles in BAT from TBT-exposed animals but not in animals exposed to DMSO or ROSI (see Supplemental Material, Figure S1). In males, we observed a trend toward reduced numbers of brown adipocytes in all three generations (only significant in F2; Figure 1C), whereas females showed strong changes in the number of cells only in F2 animals (see also Supplemental Material, Figure S2C).

*TBT exposure causes transgenerational reprogramming of MSCs to favor the adipocyte lineage*. MSCs are found in many tissues in the body, including bone marrow and adipose tissues. Depending on the stimuli they receive from surrounding cells or in culture, MSCs can differentiate into a variety of specialized cells, such as osteoblasts, adipocytes, chondrocytes, or myocytes, among others (reviewed by Pittenger et al. 1999). We previously showed that MSCs from mice exposed *in utero* to TBT were predisposed to differentiate into adipocytes at the expense of osteoblasts (Kirchner et al. 2010). To ascertain whether this effect is transmitted to the offspring of exposed animals, we analyzed the mRNA levels of a panel of adipogenic and osteogenic markers in MSCs obtained from the bone marrow of F1, F2, and F3 mice.

Early markers of enhanced adipogenic fate tested included Zfp423, a transcriptional regulator of preadipocyte determination thought to function by inducing *PPAR*γ expression (Gupta et al. 2010); PPARγ, considered to be the master regulator of adipogenesis (Tontonoz and Spiegelman 2008); and Fabp4, a preadipocyte marker whose gene is a direct PPARγ target (Burris et al. 1999; Ibrahimi et al. 1994). Pref-1 is an inhibitor of adipocyte differentiation (Sul et al. 2000) previously shown to be down-regulated by obesogen exposure in MSCs (Kirchner et al. 2010; Li et al. 2011, 2012). We also analyzed LPL, an adipogenic marker that functions to hydrolyze triglyceride bonds in lipoproteins, generating free fatty acids that can be taken up by cells and stored as triacylglycerols after esterification. LPL is also expressed in MSCs committed to the adipogenic lineage (Braun and Severson 1992).

**Figure 2:**
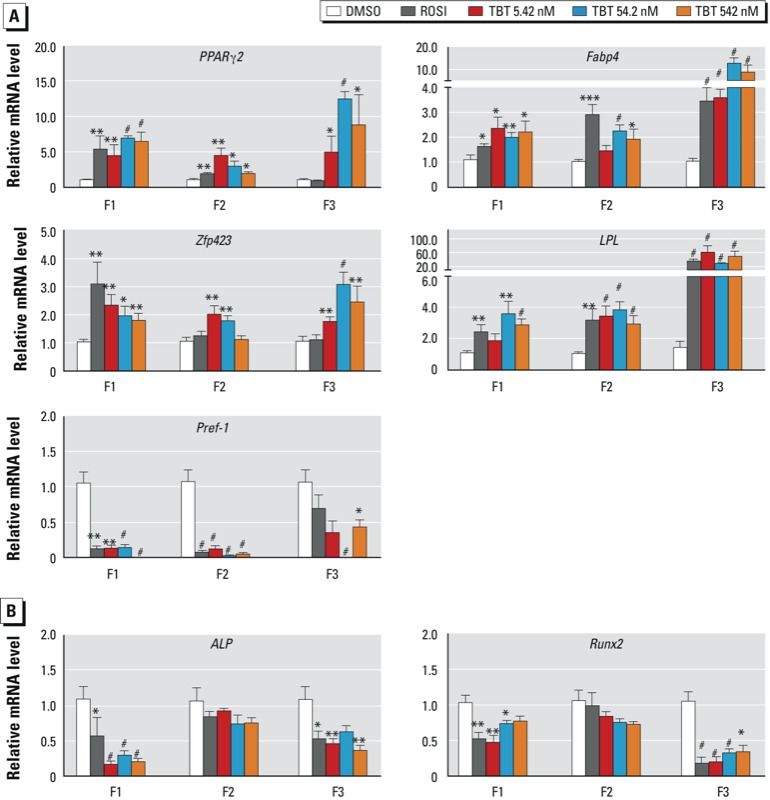
Transgenerational effects of DMSO (vehicle), ROSI, or TBT (5.42, 54.2, or 542 nM) on the gene expression profile of MSCs from F1, F2, and F3 male mice. The relative mRNA levels of specific transcripts for adipogenic (*A*) or osteogenic (*B*) differentiation were assayed by QPCR in undifferentiated MSCs, with the expression of each target gene normalized to β-actin. Data are expressed as mean fold change ± SEM of three biological replicates assayed in duplicate. *p < 0.05, **p < 0.01, and ^#^*p *< 0.001 compared with DMSO vehicle by unpaired *t*-test.

QPCR analysis of bone marrow–derived MSCs revealed sharply increased expression of adipogenic markers and decreased expression of *Pref-1* in males in the TBT exposure groups from all three generations, with the most dramatic effects in the F3 generation (Figure 2A). Data from MSCs from female mice largely followed the same trend, but the changes were less pronounced in F2 mice and somewhat variable depending on the TBT exposure group in F3 (Figure S4A). Similar to the effects seen in males, the decrease in *Pref-1* expression was most pronounced in F3 [see Supplemental Material, Figure S4A (http://dx.doi.org/10.1289/ehp.1205701)].

To address potential changes in osteogenic capacity, we analyzed the early osteogenic markers bone-specific ALP and Runx2. Expression of *ALP* and *Runx2* was sharply decreased in F1 and F3 males but unchanged in F2 (Figure 2B). MSCs from female mice showed strongly decreased *ALP* and *Runx2* expression in F1 and F3 generations and a slight decrease in F2 [see Supplemental Material, Figure S4B (http://dx.doi.org/10.1289/ehp.1205701)]. F2 males did not show a significant change in osteogenic commitment, despite the increased adipogenic commitment (Figure2B), which is not consistent with the prevailing view that commitment to fat or bone lineages is mutually exclusive (Beresford et al. 1992; Gimble et al. 2006; Takada et al. 2009). The changes observed in F3 males and females supports the contention that prenatal TBT exposure caused a transgenerational reprogramming of MSC fate to favor the adipogenic lineage at the expense of the osteogenic lineage.

**Figure 3:**
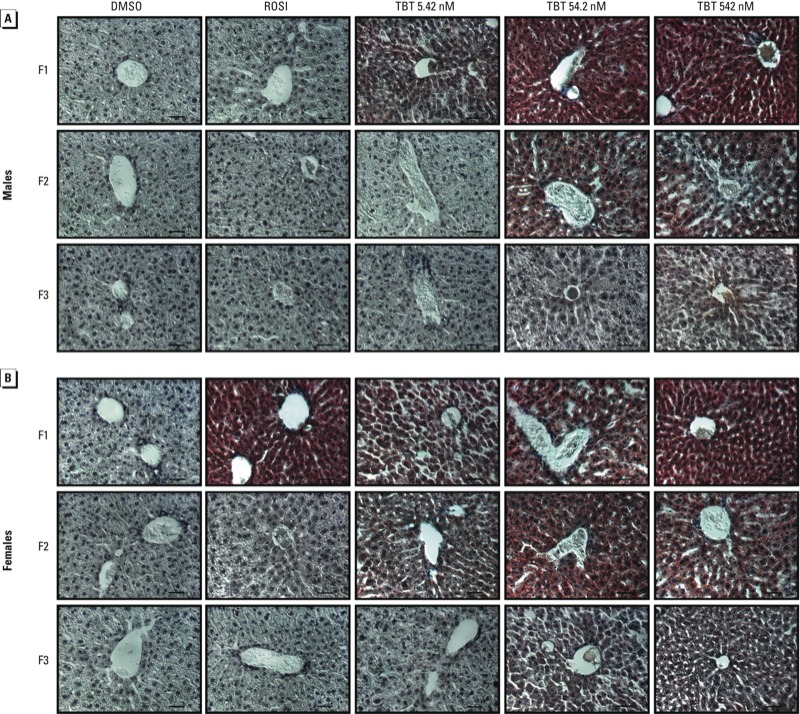
Transgenerational effects of DMSO (vehicle), ROSI, or TBT (5.42, 54.2, or 542 nM) on hepatic lipid accumulation in F1, F2, and F3 male (*A*) and female (*B*) mice. Histological sections of frozen livers were stained with Oil Red O and hematoxylin; at least five animals per exposure group were analyzed, and representative photomicrographs are shown. Bars = 50 μm.

*Prenatal TBT exposure induces fatty livers*. NAFLD is a risk factor associated with cardiovascular diseases and type 2 diabetes (reviewed by Perseghin 2011). Increased visceral adipose tissue leads to increased levels of circulating free fatty acids, which are taken up by the liver and lead to triglyceride synthesis and accumulation (Gastaldelli et al. 2007). We observed that livers from TBT-exposed F1 animals were noticeably whiter than those from controls [see Supplemental Material, Figure S5 (http://dx.doi.org/10.1289/ehp.1205701)]; therefore, we analyzed livers from all generations for lipid accumulation and the expression of genes involved in lipogenesis, lipolysis, and lipid droplet storage. We found that F1 males and females in all TBT exposure groups exhibited a pronounced increase in lipid accumulation (Figure 3). Hepatic steatosis has been observed in adult male mice exposed to TBT at puberty (Zuo et al. 2011), but this is the first report that prenatal TBT exposure can cause the same phenotype. We also observed this increased lipid accumulation in F2 and F3 generations (males and females), but it was less pronounced histologically (Figure 3). ROSI increased hepatic lipid accumulation in F1 females (Figure 3) but not in females of other generations, and ROSI elicited no changes in lipid accumulation in males of any generation.

**Figure 4:**
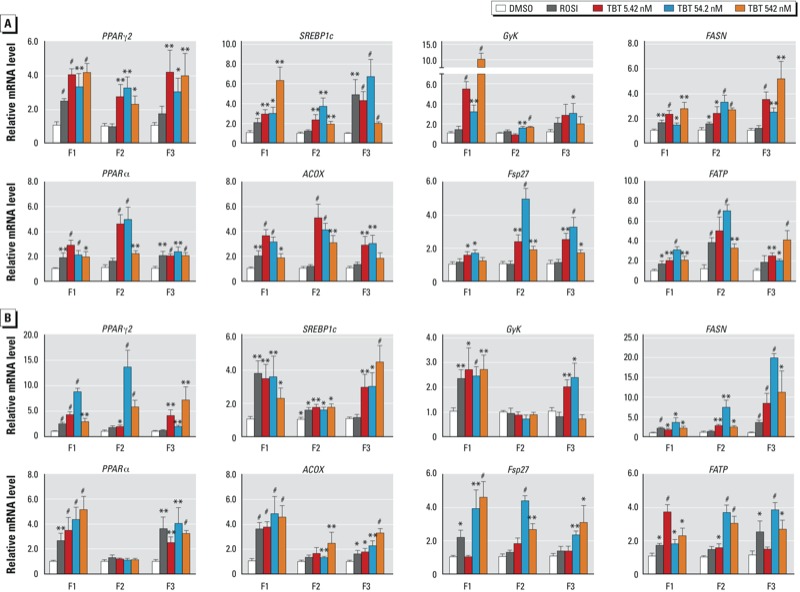
Transgenerational effects of DMSO (vehicle), ROSI, or TBT (5.42, 54.2, or 542 nM) on markers of hepatic lipid metabolism in F1, F2, and F3 male (*A*) and female (*B*) mice. Relative mRNA levels of *PPAR*γ*2*, *SREBP1c*, *GyK*, and *FASN* (lipogenic markers); *PPAR*α and *ACOX* (lipolytic markers); and *Fsp27* and *FATP* (lipid droplet markers) were evaluated in total RNA from mouse liver. Data are expressed as mean fold change ± SEM of three biological replicates assayed in duplicate. *p < 0.05, **p < 0.01, and ^#^*p *< 0.001 compared with DMSO vehicle by unpaired *t*-test.

The structure of lipid droplets is maintained by coating proteins such as Fsp27/CideC, which also participates in metabolism of the droplets (Hall et al. 2009). We analyzed hepatic mRNA levels of *Fsp27* and *FATP* and found that the expression of both is increased in TBT-exposed F1 animals and in their F2 and F3 descendants (Figure 4). To further evaluate the hepatic phenotypes, we analyzed the expression of genes implicated in hepatic lipid metabolism. Hepatic PPARγ and SREBP1c induce lipogenesis (Pettinelli and Videla 2011); FASN is a SREBP1c target that promotes fatty acid synthesis (Paulauskis and Sul 1989); and GyK is a PPARγ target that promotes glycerol uptake and lipogenesis (Tontonoz and Spiegelman 2008). PPARα and ACOX are implicated in lipolysis (Abdelmegeed et al. 2011; Reddy and Rao 2006). We observed significant increases in the expression of nearly all lipogenesis- and lipolysis-related genes in males and females from all three generations of the TBT exposure groups and in F1 ROSI-exposed animals, but males and females differed in regard to changes in *PPAR*α and *GyK* in F2 animals in the TBT groups (Figure 4). We infer from these data that prenatal TBT exposure of F1 mice has transgenerational effects on hepatic lipid metabolism that are reflected by increased hepatic fat storage in subsequent generations (F2 and F3). This is the first demonstration that prenatal obesogen exposure leads to NAFLD and that these changes can be transgenerationally inherited, which may have important implications for the increasing incidence of NAFLD.

## Discussion

Obesity and related metabolic risk factors, such as insulin resistance, hypertension, and NAFLD, have become a worldwide epidemic (Flegal et al. 2010). These risk factors are strongly associated with the subsequent development of cardiovascular disease and diabetes (de Ferranti and Mozaffarian 2008; Lusis et al. 2008; Reaven 1993). There is an urgent need to understand the mechanisms underlying the predisposition to obesity and related disorders. A growing body of evidence supports the involvement of obesogens that contribute to the burgeoning obesity epidemic (reviewed by Janesick and Blumberg 2011). It should come as no surprise that chemical obesogens exist, because a variety of pharmaceutical drugs (e.g., tricyclic antidepressants, thiazolidinedione antidiabetic drugs, atypical antipsychotics) have been associated with weight gain in humans (reviewed by Janesick and Blumberg 2011). It would be unreasonable to suppose that EDCs targeting the same pathway would not have the same effects. Indeed, we previously showed that the EDC TBT targets the same cellular pathway (PPARγ) as does the pharmaceutical obesogen ROSI, leading to weight gain *in vivo* (Grün et al. 2006) and reprogramming of MSC fate to favor the adipogenic compartment at the expense of the osteogenic fate (Kirchner et al. 2010). The fungicide triflumizole also acts through PPARγ to induce adipogenesis in MSCs and preadipocytes *in vitro* through a PPARγ-dependent mechanism and promotes increased WAT depot size and altered MSC programming *in vivo* (Li et al. 2012).

In the present study we observed that prenatal exposure of pregnant F0 animals to TBT caused transgenerational effects that increased WAT depot size, reprogrammed the fate of MSCs predisposing them to become adipocytes, and increased hepatic lipid storage and metabolism leading to apparent NAFLD in F1 and subsequent generations. These results persisted until at least the F3 generation. This suggests that the effects of prenatal obesogen exposure can be permanent, thereby leading to widespread changes at the population level over a relatively short time. Our data show the same trend for both males and females, with some relatively modest sex-specific differences. The analyses of the different adipose tissues and the gene expression profiles revealed stronger phenotypes in males than in females in all three generations. In contrast, the NAFLD-like phenotype is somewhat stronger in females than in males. In ROSI-exposed mice and their descendants, females show a more pronounced liver phenotype than do males, suggesting that there are differences in how TBT and ROSI act between tissues and sexes. Both TBT and ROSI are PPARγ activators (Grün et al. 2006; Kanayama et al. 2005), but TBT also activates the retinoid X receptor (RXR), which offers potential alternative modes of action. It will be of great interest in future studies to identify the molecular mechanisms underlying these differences.

The doses of TBT used in this study (5.42, 54.2, 542 nM) provide intakes of 0.53, 5.3, and 53 μg/kg/day respectively (assuming that a mouse weighing 30 g consumes 10 mL water/day). These intakes are, respectively, about 50-fold lower, 5-fold lower, and 2-fold higher than the established mouse NOAEL of 25 μg/kg/day (Vos et al. 1990). Moreover, they are comparable to the established human tolerable daily intake of 250 ng/kg/day, which was derived by applying a 100-fold safety factor to the mouse NOAEL (Airaksinen et al. 2010; Fristachi et al. 2009). The degree to which the tolerable daily intake has any relationship to human exposure is uncertain because, to our knowledge, there are no results from moderate- or large-scale biomonitoring studies that have established the actual TBT exposure in the population. The few available human biomonitoring studies suggest human serum concentrations of TBT in the range of approximately 27 nM (Kannan et al. 1999) and of triphenyltin at about 2 nM (Rantakokko et al. 2008). All estimates of the daily human intake of organotins fall far below the tolerable daily intake (250 ng/kg/day), which raises the important question of how the measured blood levels were acquired. Because the tolerable daily intake is based on food consumption and organotins have been found in house dust and a variety of other products (Kannan et al. 2010), human exposure may come from multiple sources, only some of which are known. Thus, the dose of TBT we used for these experiments is reasonable and biologically relevant.

It is alarming that the incidence of obesity in U.S. children is high and increasing (Koebnick et al. 2010; McCormick et al. 2010; Taveras et al. 2009), as is the incidence of NAFLD (Mencin and Lavine 2011; Widhalm and Ghods 2010). Diet and lack of exercise continue to be offered as the root cause; however, this cannot explain the results of a recent study, which showed that eight species of animals, including pets, laboratory animals, and feral rats living in proximity to humans, have become obese in parallel with the human obesity epidemic (Klimentidis et al. 2011). The likelihood of this being a chance occurrence has been estimated at about 1 in 10 million (Klimentidis et al. 2011). Although it is not impossible that each of the 24 different populations of animals examined by Klimentidis et al. (2011) has recently increased their food consumption and decreased their exercise levels, it is more reasonable to hypothesize that something else in the environment has changed. Increased exposure to environmental obesogens is one possibility. Our demonstration that prenatal exposure to TBT leads to increased adipogenesis, reprogramming of MSCs to favor adipocyte lineage, and development of fatty livers, together with the observation that these effects are passed on to the F2 and F3 generations, suggests that the effects of obesogen exposure may be even more damaging than researchers had previously thought. It will be of great interest to identify the mechanisms through which TBT exerts transgenerational effects on adipogenesis and NAFLD and the extent to which alterations in stem cell fate and function are involved.

Skinner and colleagues first showed that high doses of the fungicide vinclozolin can lead to transgenerational effects on male fertility, tumors, prostate disease, kidney diseases, and immune abnormalities (Anway et al. 2005) resulting from epigenetic changes in gene expression (Skinner 2011a). Since this landmark study, many other studies have demonstrated multigenerational effects of EDCs on a variety of organ systems (reviewed by Skinner 2011b). Although early studies showed transgenerational inheritance of EDC effects elicited by high doses of single chemicals, more recent studies showed that high doses of a variety of chemicals (fungicides, pesticide mixture, plastic mixture, dioxin, and a hydrocarbon mixture) could also elicit transgenerational effects (Manikkam et al. 2012; Nilsson et al. 2012). Wolstenholme et al. (2012) recently showed that administration of bisphenol A at a dose that achieved plasma levels similar to those measured in humans could cause transgenerational alterations in genes and behavior. Ours is the first study to demonstrate transgenerational effects of developmental exposure to environmentally relevant concentrations of an obesogen on adipogenesis, MSC programming, and hepatic fat accumulation. These results have important implications both for understanding the obesity epidemic and for the ongoing discussion about the dangers of EDCs. If, as we expect, our model applies to humans, then prenatal obesogen exposure could permanently reprogram the metabolism of exposed individuals, predisposing them toward weight gain, particularly in conditions of caloric excess. The transgenerational inheritance of obesogen exposure, particularly the increased magnitude of the effects in the F3 generation, raises the stakes in the ongoing debate regarding what weight of evidence is required for regulatory agencies to take action to reduce EDC exposure.

## Supplemental Material

(4.7 MB) PDF
