# Supplementary material for: Transgenerational Inheritance of Increased Fat Depot Size, Stem Cell Reprogramming, and Hepatic Steatosis Elicited by Prenatal Exposure to the Obesogen Tributyltin in Mice
Source: Environ Health Perspect. 2013 Jan 15;121(3):359–66. doi: 10.1289/ehp.1205701 (PMC3621201; doi:10.1289/ehp.1205701)
Supplement: (4.7 MB) PDF [file ehp.1205701.s001.pdf]

Supplemental Material

Transgenerational Inheritance of Increased Fat Depot Size, Stem Cell Reprogramming, and Hepatic Steatosis Elicited by Prenatal Obesogen Tributyltin in Mice

Raquel Chamorro-García, Margaret Sahu, Rachelle J.Abbey, Jhyme Laude, Nhieu Pham and Bruce Blumberg[2]

Table of Contents

Table S1. Number of litters per group 2

Table S2. Number of animals per group 3

Table S3. Primers used for QPCR analysis of gene expression 4

Figure S1. Hematoxylin and eosin staining of sections of epididymal, peri-renal, interscapular white and interscapular brown adipose tissues from F1 males. 5

Figure S2. Transgenerational effects of TBT on ovarian, perirenal, subscapular white and subscapular brown adipose tissues from F1, F2 and F3 female mice. 6

Figure S3. Transgenerational effects of TBT on the body weights of F1, F2 and F3 mice.......... 7

Figure S4. Gene expression profiles of MSCs from F1, F2 and F3 female mice.......... 8

Figure S5. Macroscopic phenotype of livers from F1 males and females. 9

Table S1. Number of litters per group

	DMSO	ROSI	TBT 5.42 nM	TBT 54.2 nM	TBT 542 nM
F1	5	4	4	4	4
F2	5	4	4	5	4
F3	4	4	4	4	4

Table S2. Number of animals per group

		DMSO	ROSI	TBT 5.42 nM	TBT 54.2 nM	TBT 542 nM
MALES	F1	16	10	11	11	11
	F2	12	10	10	14	10
	F3	11	15	14	10	11
FEMALES	F1	16	10	10	12	14
	F2	16	10	10	11	10
	F3	10	9	8	11	11

Table S3. Primers used for QPCR analysis of gene expression

Gene	Forward	Reverse
ACOX	CAGGAAGAGCAAGGAAGTGG	CCTTTCTGGCTGATCCCATA
ALP	GGGACTGGTACTCGGATAACGA	CTGATATGCGATGTCCTTGCA
β-actin	GGCTGTATTCCCCTCCATCG	CCAGTTGGTAACAATGCCATGT
Fabp4	AGCCCAACATGATCATCAGC	TTTCCATCCCACTTCTGCAC
FATP	GGCGTTTCGATGGTTATGTT	CAGCTCGTCCATCACTAGCA
Fsp27/CideC	CTGGAGGAAGATGGCACAAT	GGGCCACATCGATCTTCTTA
FASN	GTGTCCAAGAAGTGCAGCAA	GGAGCGCAGGATAGACTCAC
GyK	AATCCGCTGGCTAAGAGACAACCT	CCCTGAAAATGCTGGAACGAAGTA
LPL	ACAACCAGGCCTTCGAGATT	TCAGGCCAGCTGAAGTAGGA
PPARα	TTTCCCTGTTTGTGGCTGCTA	CCCTCCTGCAACTTCTCAATG
PPARγ	TGGGTGAAACTCTGGGAGATTC	AATTTCTTGTGAAGTGCTCATAGGC
Pref-1	CCTGGCTGTGTCAATGGAGT	CTTGTGCTGGCAGTCCTTTC
Runx2	TTTAGGGCGCATTCCTCATC	TGTCCTTGTGGATTAAAAGGACTTG
SREBP1	ACGAAGTGCACACAAAAGCA	GCCAAAAGACAAGGGGCTAC
Zfp423	TGGACCACCGTGATCTTACA	TCGTTCTCGAACGTCATCTG

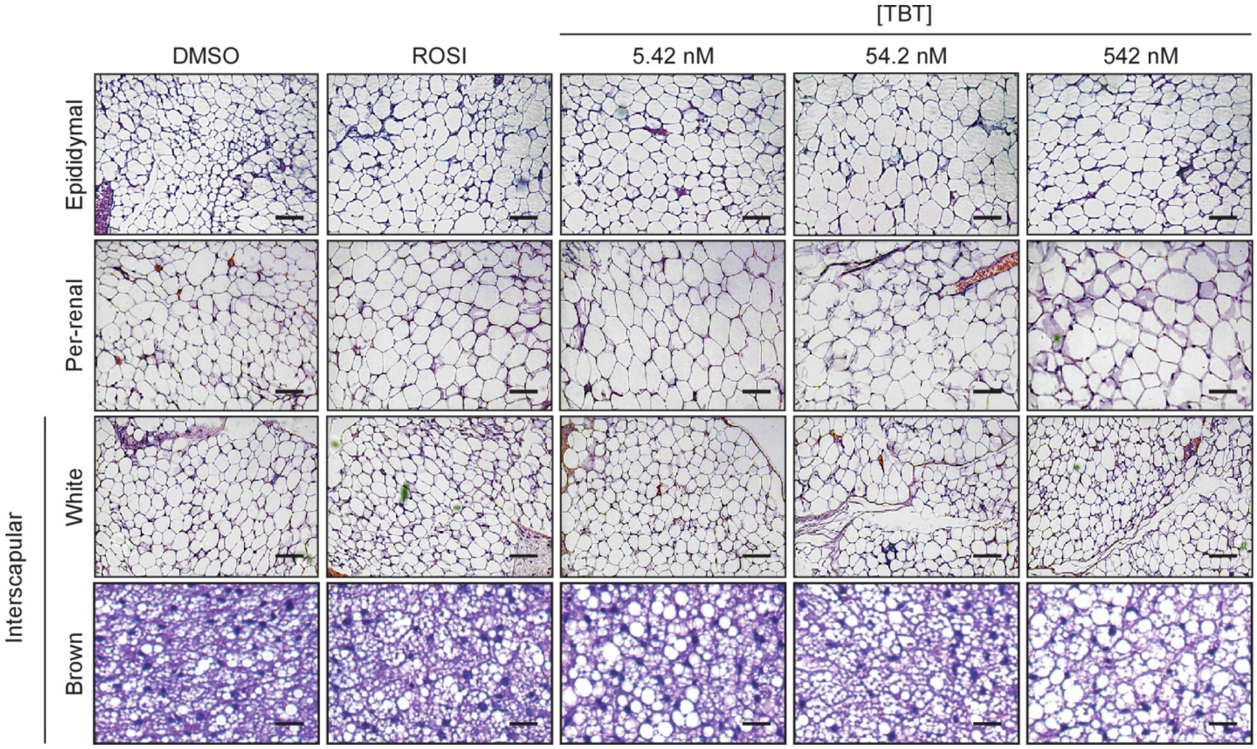

Figure S1. Hematoxylin and eosin staining of sections of epididymal, peri-renal, interscapular white and interscapular brown adipose tissues from F1 males.

Adipose depots were dissected, fixed with buffered 3.7% formaldehyde and then embedded, sectioned and stained with hematoxylin and eosin using standard methods in the UCI Pathology Core facility. The bar represents 50 μm in each panel.

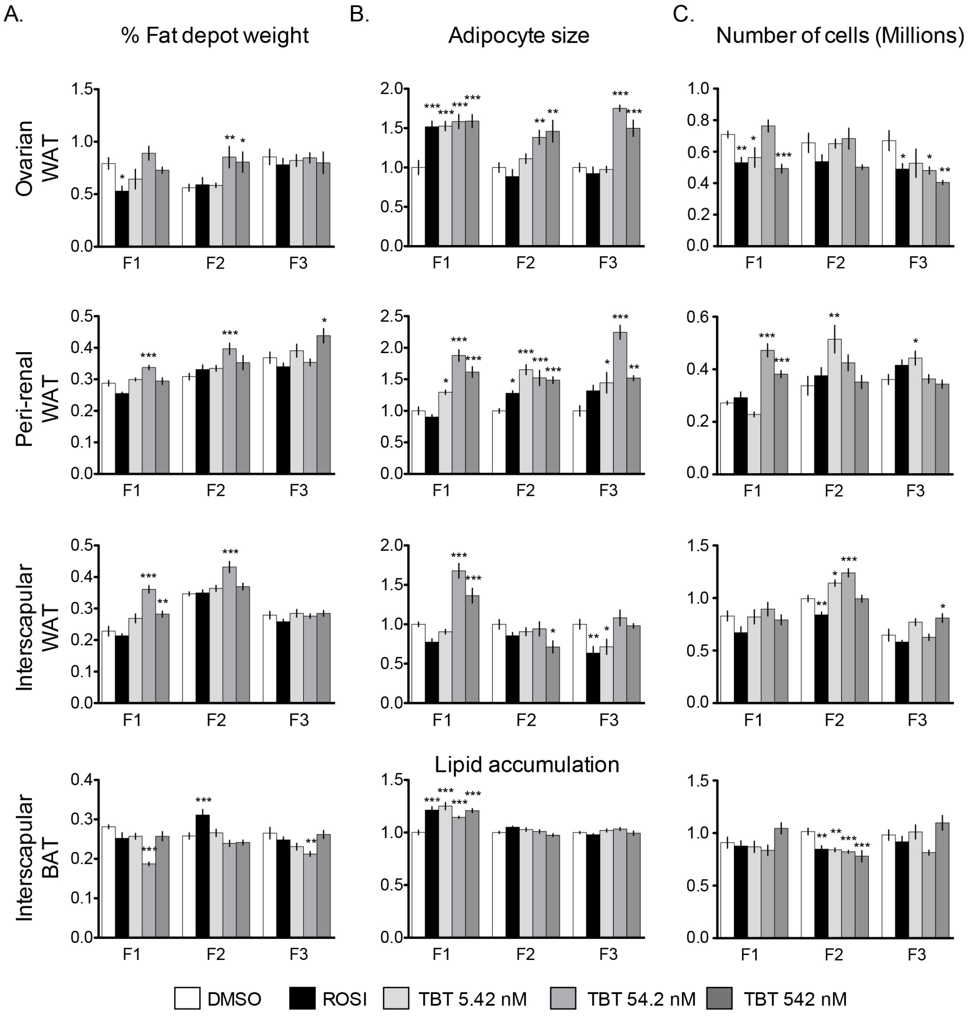

Figure S2. Transgenerational effects of TBT on ovarian, perirenal, subscapular white and subscapular brown adipose tissues from F1, F2 and F3 female mice.

A) Adipose tissue weights were represented as the percentage of total body weight. B) Relative adipocyte size in ovarian-, perirenal- and subscapular white- adipose tissues, and lipid accumulation for subscapular brown adipose tissue. C) Number of adipocytes per fat depot assayed by total DNA quantitation. All data are expressed as averages from 8-16 animals ± SEM. For statistical analyses, samples were assayed with one-way ANOVA followed by Dunnett's posthoc test. *, $P<0.05$; **, $P<0.01$; ***, $P<0.001$.

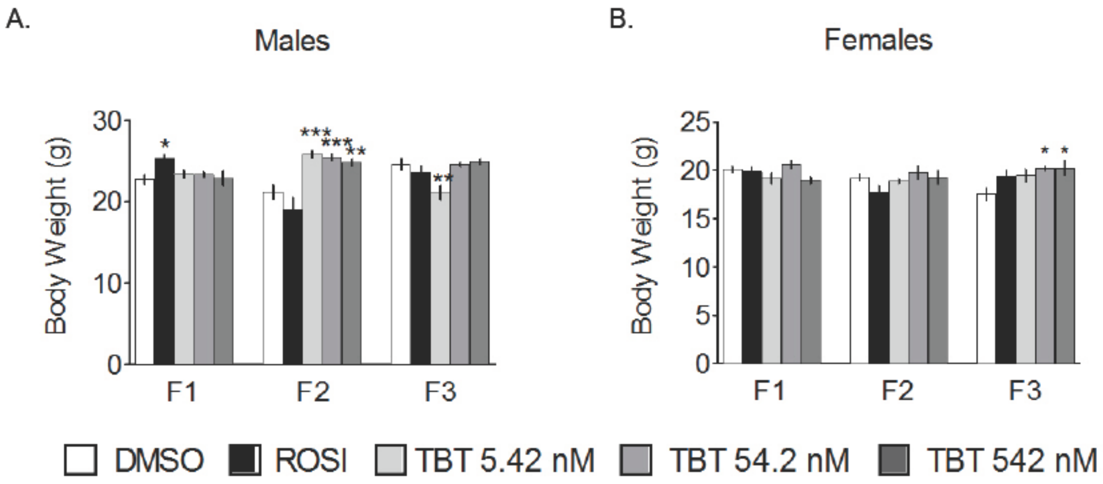

Figure S3. Transgenerational effects of TBT on the body weights of F1, F2 and F3 mice.

Total body weight was measured after 4 hours of fasting prior to euthanasia. A) male and B) female mice. For statistical analyses, samples were assayed with one-way ANOVA followed by Dunnett's posthoc test. *, $P<0.05$; **, $P<0.01$; ***, $P<0.001$.

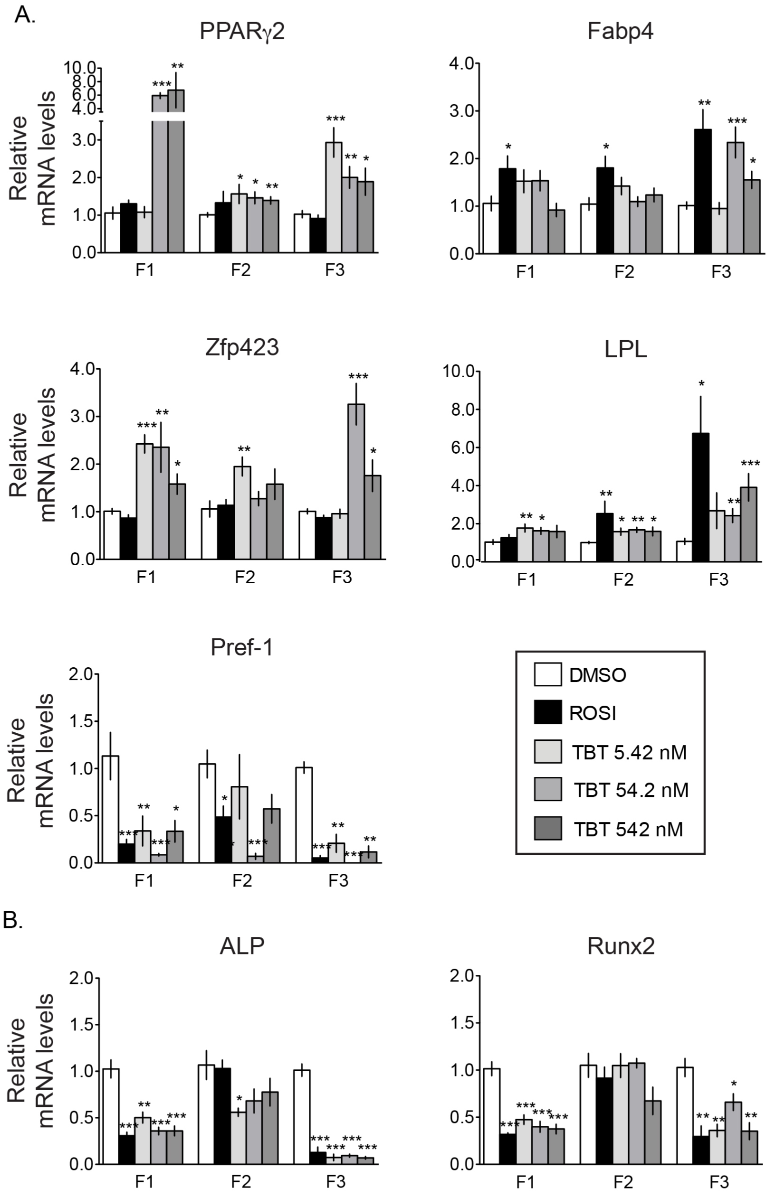

Figure S4. Gene expression profiles of MSCs from F1, F2 and F3 female mice.

The relative mRNA levels of specific transcripts for adipogenic (A) or osteogenic (B) differentiation were assayed by qPCR in undifferentiated MSCs, with the expression of each target gene normalized to β-Actin. All data is expressed as average fold change ± SEM in 3 biological replicates run in duplicate. We used unpaired t-tests for statistical analyses. *, $P<0.05$; **, $P<0.01$; ***, $P<0.001$.

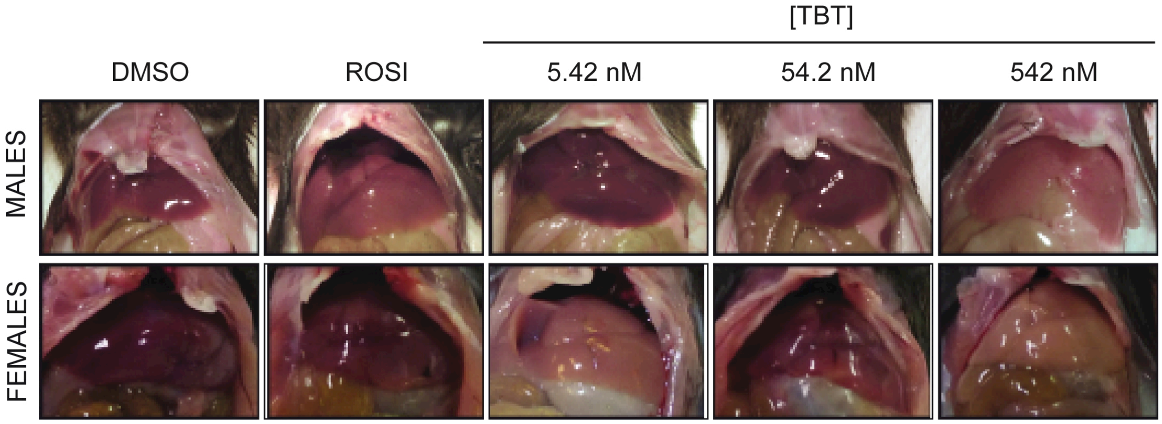

Figure S5. Macroscopic phenotype of livers from F1 males and females.

Livers of representative animals were photographed during dissection to illustrate the pale color of livers in treated animals.
